# Social Media Addiction as a Mediator in the Personality–Performance Relationship

**DOI:** 10.1192/j.eurpsy.2026.10823

**Published:** 2026-07-31

**Authors:** N. Rmadi, R. Affes, N. Kotti, F. Dhouib, M. Hajjaji, K. Jmal Hammami

**Affiliations:** 1 Occupational medicine department, Hedi Chaker university hospital; 2 Family medecine department, Faculty of medecine of Sfax, Sfax, Tunisia

## Abstract

**Introduction:**

The widespread use of social media among young physicians raises concerns about its impact on professional efficiency. This study examined whether social media addiction mediates the relationship between personality traits and job performance.

**Objectives:**

To evaluate the mediating role of social media addiction in the relationship between personality traits and professional performance in medical residents.

**Methods:**

A total of 429 medical residents completed three standardized questionnaires: the BFI-20, assessing five personality traits; the BSMAS, measuring social media addiction; and the IWPQ, evaluating work performance across task performance, contextual performance, and counterproductive behavior. Mediation models tested whether personality traits indirectly influenced work performance through social media addiction.

**Results:**

Social media addiction significantly reduced the positive effects of several personality traits on job performance. Indirect effects were strongest for extraversion (β=–0.302, p<0.001) and openness (β=–0.296, p<0.001). Conscientiousness, normally a strong predictor of higher performance, was also weakened by social media addiction (β=–0.141, p=0.002).

**Conclusions:**

Social media addiction systematically undermines the protective role of personality traits on professional outcomes. Addressing excessive use of social networks is crucial for maintaining the benefits of personality resources in residency training.

**Disclosure of Interest:**

None Declared

